# Deep learning in photoacoustic imaging: a review

**DOI:** 10.1117/1.JBO.26.4.040901

**Published:** 2021-04-09

**Authors:** Handi Deng, Hui Qiao, Qionghai Dai, Cheng Ma

**Affiliations:** a Tsinghua University, Department of Electronic Engineering, Haidian, Beijing, China; b Tsinghua University, Department of Automation, Haidian, Beijing, China; c Tsinghua University, Institute for Brain and Cognitive Science, Beijing, China; d Tsinghua University, Beijing Laboratory of Brain and Cognitive Intelligence, Beijing, China; e Tsinghua University, Beijing Key Laboratory of Multi-Dimension and Multi-Scale Computational Photography, Beijing, China; f Beijing Innovation Center for Future Chip, Beijing, China

**Keywords:** photoacoustic imaging, deep learning, convolutional neural network

## Abstract

**Significance:** Photoacoustic (PA) imaging can provide structural, functional, and molecular information for preclinical and clinical studies. For PA imaging (PAI), non-ideal signal detection deteriorates image quality, and quantitative PAI (QPAI) remains challenging due to the unknown light fluence spectra in deep tissue. In recent years, deep learning (DL) has shown outstanding performance when implemented in PAI, with applications in image reconstruction, quantification, and understanding.

**Aim:** We provide (i) a comprehensive overview of the DL techniques that have been applied in PAI, (ii) references for designing DL models for various PAI tasks, and (iii) a summary of the future challenges and opportunities.

**Approach:** Papers published before November 2020 in the area of applying DL in PAI were reviewed. We categorized them into three types: image understanding, reconstruction of the initial pressure distribution, and QPAI.

**Results:** When applied in PAI, DL can effectively process images, improve reconstruction quality, fuse information, and assist quantitative analysis.

**Conclusion:** DL has become a powerful tool in PAI. With the development of DL theory and technology, it will continue to boost the performance and facilitate the clinical translation of PAI.

## 1 Photoacoustic Imaging

### 1.1 Brief Introduction to Photoacoustic Imaging

Photoacoustic imaging (PAI), also referred to as optoacoustic imaging, is an emerging imaging technique that works in both the optically ballistic and diffusive regimes. In addition to providing good contrast of blood vessels,^1^ PAI is also capable of functional (such as blood oxygen saturation, ${\mathrm{sO}}_{2}$) and molecular imaging.^2^^,^^3^

PAI relies on the photoacoustic (PA) effect, which was first discovered by Alexander Graham Bell in 1880 as a conversion of light intensity modulation into sound emission.^4^ The rapid development of PAI in the past 30 years was incentivized by the maturation of laser and ultrasound (US) technologies. In a common PAI setting, when biological tissue is illuminated by a short optical pulse, a local temperature rise is induced, which in turn produces ultrasonic waves. Acoustic detectors outside the tissue receive the PA signal and an image is subsequently reconstructed digitally to visualize the initial pressure rise distribution (which is closely related to the optical absorption distribution).

### 1.2 Image Formation

PAI can be categorized into two types according to the image formation principle. The first is photoacoustic microscopy (PAM),^5^ which is based on raster-scanning a focused ultrasonic transducer or a beam of focused light, to acquire an image pixel by pixel (each pixel is an A-line in the longitudinal direction; resolution along the A-line is generated by acoustic delay). In this implementation, the received PA signal mainly comes from the focal line, thus all spatial information is unmixed and no reconstruction is needed. The second type is photoacoustic computed tomography (PACT),^6^ in which signals are detected by a multi-element transducer array (or by scanning a single unfocused transducer). Each transducer element has a large acceptance angle within the field of view, and a PA image can be reconstructed by merging the data from all transducer elements. Popular arrays used in PACT include linear,^7^^,^^8^ ring (and partial ring),^9^^,^^10^ spherical (and partial spherical), and planar arrays.^11^^,^^12^ Linear arrays are easy to operate and relatively cheap, thus they were widely used for clinical applications. However, their angular acceptance is limited, resulting in poor image quality. In contrast, ring and spherical arrays have wider signal acceptance, and typically they generate better images. Yet, due to geometrical confinement, these arrays are often used for breast imaging and animal imaging and are relatively expensive. In all cases, the US transducers are limited in temporal bandwidth. As a result, deprived of a significant portion of spatiotemporal information, PAI is in constant need of better image reconstruction, processing, and analysis methods.

Traditional methods for PACT image reconstruction include Fourier domain method,^13^^,^^14^ filtered back-projection,^15^ delay and sum,^2^ time reversal,^16^^,^^17^ and model-based method.^18^^,^^19^ For details about these methods, readers are referred to the review papers in Refs. 20 and 21. To recover an image with high fidelity, all existing image reconstruction methods demand wide coverage (preferably 4π solid angle), dense spatial sampling, and broadband temporal response. Such stringent requirements impose tremendous challenges to the US detection hardware, and no imaging system has met all these requirements to date, due to various technical and economic constraints. The resultant image quality reduction and poor quantification have become major roadblocks as PAI is being pushed into the clinics. Traditional image reconstruction methods have shown limited performance in recovering the lost image information under non-ideal detection. One major motivation for introducing deep learning (DL) into PAI is to improve image quality. Moreover, DL can provide a viable means to accelerate the speed of computation.

### 1.3 Quantitative PAI

The goal of quantitative PAI (QPAI) is to image concentrations of chromophores in tissue, thereby important physiological information can be inferred.^22^ For example, blood oxygen saturation is a biomarker of tumor malignancy.^23^ The distinct spectral features of oxy- and deoxyhemoglobin in the near-infrared allow measurement of their concentration ratio by spectral unmixing, enabling the quantification of oxygen saturation.^24^ In PAI, the initial pressure is a product of the local absorption coefficient, the local Gruneisen coefficient, and the local light fluence,^25^ i.e.,

$$p_{o}(x,\lambda )=\Gamma (x)\phi (x,\lambda )\mu_{a}(x,\lambda ),$$ (1)

where x and λ denote the spatial position and the illumination wavelength, respectively, Γ(x) is the Gruneisen coefficient, which is a thermodynamic property of tissue, ϕ(x,λ) is the wavelength-dependent light fluence, and $\mu_{a}(x,\lambda )$ is the optical absorption coefficient to be determined in QPAI. Since ϕ(x,λ) and $\mu_{a}(x,\lambda )$ are globally coupled [any local change of $\mu_{a}(x,\lambda )$ tends to alter ϕ(x,λ) globally], QPAI is a highly non-linear and complex optical inversion problem. Traditional forward light propagation models include the radiative transfer equation and its approximation (e.g., diffusion approximation and δ Eddington approximation) and the Monte Carlo (MC) method for light transport.^22^ Existing methods for conducting optical inversion in PAI to decouple the local fluence and absorption coefficient include linearization, direct inversion, fixed-point iteration, eigenspectra decomposition, and model-based minimization.^22^^,^^26^ Nevertheless, the accuracy, robustness, and efficiency of these methods need further validation. In recent years, DL has shown great potential in solving QPAI problems.

## 2 Deep Learning

### 2.1 DL and Convolutional Neural Networks

DL is a set of methods that utilize multiple processing layers to discover intricate structures in high-dimensional data and has made great impact in computer vision,^27^ natural language processing,^28^ knowledge graph,^29^ and medical image analysis.^30^^,^^31^

In DL, the data set used to train the network determines the generalization ability and robustness of the learning model. As a result, data set construction is always a key issue in DL. Loss function (LF) is used to measure how well the network completes the task during training. For image processing, mean square error (MSE) is the earliest and most widely applied LF. Later, some metrics for the evaluation of image quality, such as structural similarity index measure (SSIM) and peak-signal-to-noise ratio (PSNR), were introduced as loss terms. Popular architectures for DL include stacked autoencoders, deep Boltzmann machines, recurrent neural networks (RNN), and convolutional neural network (CNN). Among them, CNN is the most commonly used model in computer vision and image processing and has been studied extensively in PAI.^32^ A typical CNN architecture contains subsequent layers of convolution, pooling, activation, and classification (full connection). The convolution layer generates the feature graph by convolving the kernel with the input. Activation functions determine the output of some layers (e.g., convolution layer and full connection) by introducing non-linearity. Common activation functions are sigmoid, tanh, rectified linear unit (ReLU), and ReLU’s variants such as Leaky. ReLU is one of the most commonly used activation functions because of its strong non-linearity and the ease of gradient calculation. The classification layer is generally a fully connected layer, which is used to connect the feature map and the output. Normalization layers can be added between the convolutional layers and the activation functions to speed up the training process and reduce the susceptibility to network initialization. It applies a transformation that maintains the mean activation close to zero and the activation standard deviation close to one. The most commonly used normalization method is batch normalization.

**Fig. 1:**
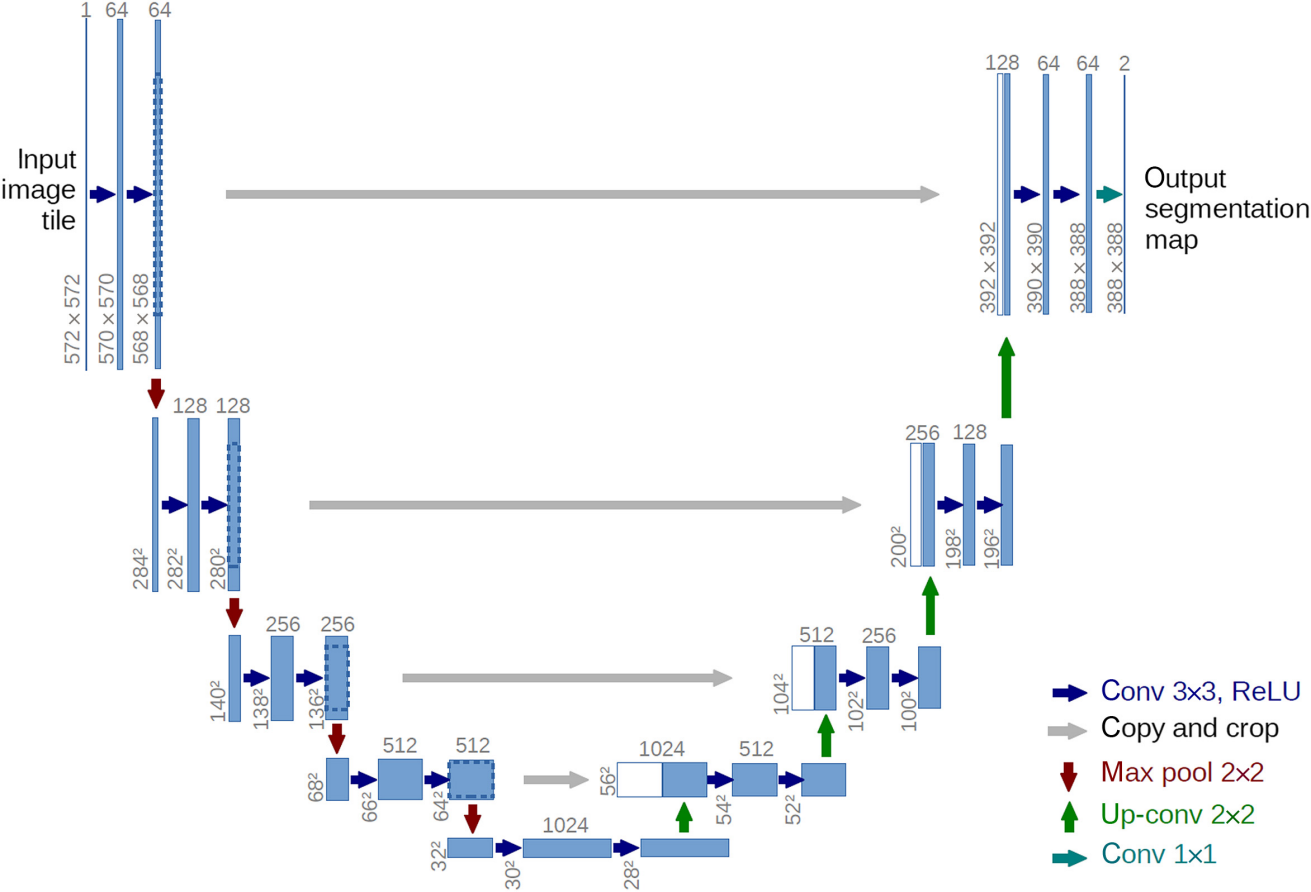
The architecture of U-Net.^34^ Each blue box corresponds to a multi-channel feature map. White boxes represent copied feature maps. Gray arrows indicate straight—connected path on the same level.

So far, many architectures of CNN have been proposed. Lee et al.^33^ summarized those that were widely used in medical image processing. One popular architecture is U-Net, proposed by Long and Shelhamer.^34^ In PAI, many published works were based on this architecture or its variants.^35^^–^^37^ The basic U-Net architecture is shown in Fig. 1. The architecture consists of a contracting path to capture context and a symmetric expanding path that enables precise localization. This allows the network to effectively extract image features at different levels with outstanding performance for end-to-end tasks. The copy and crop paths (skip connections) make the network more robust and can achieve better performance with fewer data.

### 2.2 Tools

#### 2.2.1 Popular DL tools

With the rapid hardware and software development, DL is being rapidly implemented in various fields. Popular open-source frameworks include: Caffe, Tensorflow, Theano, MXNet, and Torch7/PyTorch/PyTorch2 and Caffe2.^38^^,^^39^ Many of these frameworks provide multiple interfaces, such as C/C++, MATLAB, and Python. In addition, several packages provide advanced libraries written on top of these frameworks, such as Keras. These tools enabled the widespread applications of DL in various fields.

#### 2.2.2 Tools for building data set

Since PAI has not been widely applied in the clinics, there are currently insufficient data sets for deep neural network training. Consequently, common remedies to this problem include using simulated data for proof-of-concept verification, or training the network before conducting transfer learning with real experimental data.

The PA effect involves a light scattering process followed by an US propagation process. To simulate US propagation, the k-space pseudo-spectral method can be used.^40^ It combines the calculation of spatial derivatives (such as the Fourier collocation method) with a temporal propagator expressed in the spatial frequency domain or k-space. k-wave is an open source acoustics simulation toolbox for MATLAB and C++ developed by Treeby and Cox.^41^ It provides great flexibility and functionality in PA simulation and can be used for time reversal-based image reconstruction.^42^ Alternatively, one can solve the acoustic propagation equation using second order finite-difference time-domain (FDTD) method or finite-element method,^43^^,^^44^ to this end, COMSOL can be used.^45^

To simulate the light scattering process or to perform model-based spectral unmixing in QPAI, the MC method or radiative transfer equation can be implemented as the forward model (see Sec. 1.3). The MC method is considered to be the gold standard.^46^ It is also compatible with parallel processing to reduce computational time. mcxyz is a GPU-accelerated MC simulation tool to calculate light transport in optically heterogeneous tissues.^47^ MCXLAB is a MATLAB package that implements the MC model. Alternatively, the radiative transfer equation or its approximation can be implemented as the forward model. For example, assuming near-diffused propagation of light dramatically reduces the need for computing power, thus fluence can be calculated numerically by FDTD or finite-element method.^46^ Nirfast is an open source software that allows users to easily model near-infrared light transport in tissue.^48^ COMSOL is a multi-physics simulation tool based on finite-element analysis, which can also be used to calculate light fluence distribution.^45^

In PAI, currently reported training data sets have been listed in Table 1. Most of them contain MRI and x-ray CT images that can be transformed into PA images by simulation, whereas the last one provides real PA images. Detailed information about the construction of numerical phantoms for PAI can be found in Ref. 61. DL is also used for segmenting different tissue types when unlabeled data from other imaging modalities are applied for simulating PA raw data.^62^ In addition, to improve preclinical image quality and accelerate PAI’s clinical translations, the international photoacoustic standardization consortium is pushing forward an open-access platform for standardized PA reference data.^63^

**Table 1:** Data sets commonly used in DL-based PAI

Data set	Descriptions
Mammography image database from LAPIMO EESC/USP	The database consisted of around 1400 screening mammography images from around 320 patients.^49^
DRIVE dataset	The database was used for comparative study of vascular segmentation in retinal images. It consisted of 40 photographs, 7 of which showed signs of mild early diabetic retinopathy.^50^
Optical and acoustic breast database (OA-breast)	The database includes a collection of numerical breast phantoms generated from clinical magnetic resonance angiography data collected from Washington University in St. Louis School of Medicine.^51^
Digital mouse	The database includes a 3D whole body mouse atlas from coregistered high-quality PET x-ray CT and cryosection data of a normal nude male mouse.^52^^,^^53^
Shepp–Logan phantom	The Shepp–Logan phantom is a standard test image created by Larry Shepp and Benjamin F. Logan for their 1974 paper “The Fourier Reconstruction of a Head Section.”^54^
3D volume of CBA mouse brain vasculature	The database includes a high-resolution volumetric and vasculature atlas on CBA mouse brain based on a combination of magnetic resonance imaging and x-ray CT.^55^
ELCAP public lung image database	The database consists of an image set of 50 low-dose whole-lung CT scans. The CT scans were obtained in a single-breath hold with a 1.25-mm slice thickness. The locations of nodules detected by the radiologist are also provided.^56^
Big data from CT scanning	CT scans of several cadavers are provided. The data are collected at Massachusetts General Hospital at multiple different radiation dose levels for different x-ray spectra and with representative reconstruction techniques.^57^
Tumor phantom in mouse brain	The database is based on segmentation of a micro-CT scan of a mouse brain into gray mater, vasculature, and dura mater. An artificial cancer tissue was created by a stochastic growth process. https://github.com/asHauptmann/3DPAT_DGD/tree/master/phantomData.
VICTRE project	A series of toolkits include breast Phantom, breastCompress, and breastCrop. Using breast Phantom, the digital breast with varying patient characteristics (breast shape, glandularity and density, and size) can be generated. https://github.com/DIDSR/VICTRE.
CBIS-DDSM (curated breast imaging subset of DDSM)	The data set contains 2620 scanned film mammography studies including normal, benign, and malignant cases with verified pathology information.^58^^,^^59^^,^^60^
Mouse PACT^37^	The database consists of six athymic nude-Fox1nu mice (Harlan Laboratories) *in vivo* PA images. Each mouse was scanned over 50 mm in 0.5 mm steps, with a total of 100 cross-sectional images covering the full torso from the shoulders to the lower abdomen. https://github.com/ndavoudi/sparse_artefact_unet/blob/master/dataset/getdata.sh.

## 3 Applications of DL in PAI

Currently, applying DL in PAI has been extensively studied, and there are already several review papers. In the review by Yang et al.,^64^ they used a schematic diagram to describe the relationship between PAI tasks and the network architectures. Several open sources for implementing DL in PAI were also listed. Andreas and Ben^65^ introduced the basic principles of DL and the PA image reconstruction methods. They trained and tested the DL models with different architectures on the same data set and demonstrated and compared their performance. Gröhl et al. summarized and provided detailed statistics of the existing works in Ref. 66. Key insights for each type of work were given from the authors’ perspective. This review aims at linking the technical difficulties in PAI and the DL methods that are suitable to address them. We hope to help readers understand how to build data sets and design suitable networks for their specific tasks.

The applications of DL techniques in PAI can be classified into three types of tasks: image understanding, PA initial pressure reconstruction, and quantitative PA imaging. Tasks of the first type involve using DL for PA image classification and segmentation. It also involves image registration in various anatomical structures and tissues. This type of work is covered in Sec. 3.1. Tasks of the second type focus on improving image quality under non-ideal detection conditions, where common non-ideal conditions include: limited bandwidth, sparse spatial sampling, and limited-view detection. In Sec. 3.2, we first talk about image reconstruction under a single-non-ideal detection condition. Then how DL can be used to alleviate image degradation associated with non-uniform speed of sound (SoS) will be introduced. Next, the complex scenario when multiple non-ideal conditions coexist will be discussed, such situations are more relevant to real applications. In this case, DL can be used to reconstruct PA images with better fidelity, irrespective of the specific type of the information deficiency. Finally, we introduce how DL can be used in conjunction with economical and portable PAI systems, such as single-channel data acquisition (DAQ) and light-emitting diode (LED)-based systems. For the last type of tasks, DL is used to improve the quantification accuracy and calculation speeds in QPAI. These works will be discussed in Sec. 3.3, where we first introduce how DL helps the calculation of ${\mathrm{sO}}_{2}$, chromophore concentration, and fluence spectra, then we show that DL can be used to select regions, in which QPAI calculation is reliable (which is in fact a segmentation task).

**Fig. 2:**
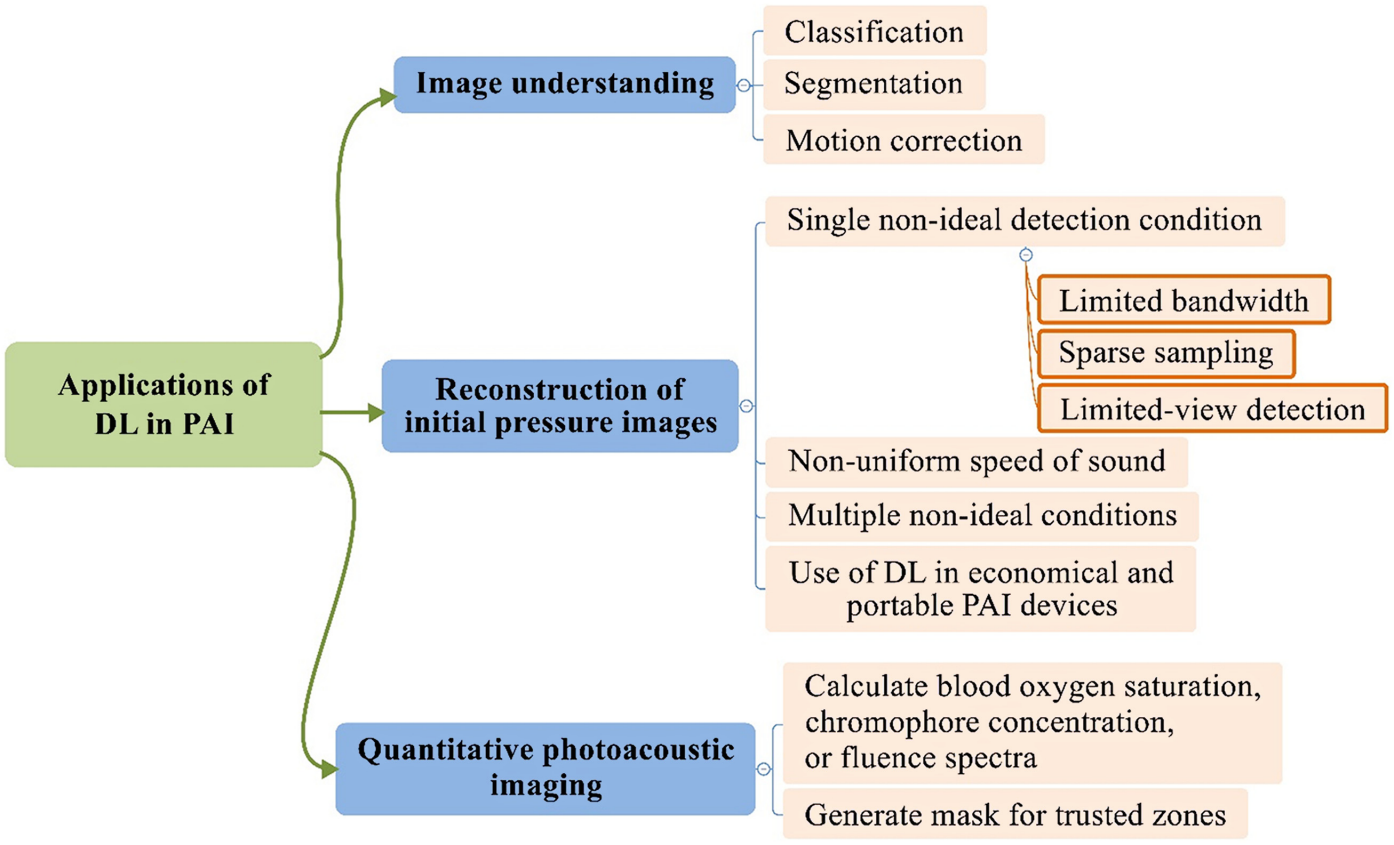
Diagram showing the applications of DL in PAI, and the structure of this review follows this diagram.

Figure 2 summarizes the structure of the above classification, which also highlights the organization of this review. Most of the references cited in this review were searched in Google Scholar with keywords “DL” and “PA”, “DL” and “optoacoustic,” “neural network” and “PA,” “neural network” and “optoacoustic,” “machine learning” and “PA,” “machine learning” and “optoacoustic”, and we tried not to miss important works by checking all relevant precursors of the original paper pool. So this review should cover DL-PAI works published before November 2020.

### 3.1 Image Understanding

Image classification and segmentation are basic tasks in image understanding. Using DL to do auxiliary diagnosis and segmentation of lesion regions on PA images appeared in 2012.^67^ Unlike traditional image segmentation, segmentation of PA images can be seen as a special reconstruction task and achieved a great result.^68^ Table 2 shows the PA image understanding tasks and the networks applied. Here NN denotes “neural network” and “simple NN/CNN” refers to a NN/CNN without a complex or specific architecture, such as squeeze-and-excitation (SE)-blocks, residual layer, or dilated convolution kernel; “complex NN/CNN” refers to a NN/CNN with complex or specific architecture but without a well-known name. U-Net and its variants are called U-Net. The above naming rules apply to Tables 3 and 4 as well.

**Table 2:** Network architectures used in PA image understanding.

General task	Specific task	Network architecture
Image understanding	Classification	Simple NN;^67^^,^^69^ AlexNet;^70^ GoogLeNet;^70^ Resnet;^71^^,^^72^ Simple CNN;^73^^,^^74^ simple CNN combined with traditional classifier;^74^^,^^75^ and ResNet18^76^
	Segmentation	U-Net^77^^,^^78^ and simple NN applied in iterative method^68^
	Motion correction	Simple CNN^79^

Auxiliary diagnosis is one of the most common applications of DL. As early as 2012, Alqasemi et al.^67^ utilized a neural network as a classifier to facilitate ovarian cancer diagnosis on US and PACT images. In this work, features for classification were extracted from PA images by traditional methods (Fourier transform, image statistics, and composite image filters). Its performance was not as good as support vector machine (SVM). In 2016, Rajanna et al.^69^ used more features from the time and frequency domains of PAM images and applied three commonly used activation functions to classify prostate cancer into three classes (malignant, benign, and normal), achieving 95.04% accuracy on average. In these works, the authors used only a simple NN as the classifier, rather than extracting features by using convolution layers or other deeper network architectures. Consequently, the NN did not exhibit superiority over traditional methods.

In 2018, Zhang et al.^70^ applied AlexNet and GoogLeNet to classify breast cancer on stimulated PACT data. They achieved accuracies of 87.69% and 91.18% using AlexNet and GoogLeNet, respectively. In comparison, the accuracy of SVM was only 82.14%. Data augmentation and transfer learning were used to tackle data deficiency. They resized the images to fixed sizes and took steps to amplify the data set on preprocessing, including scaling, cropping, and random changing of the pixel value and contrast. The authors used pretrained AlexNet and GoogLeNet, and only modified and retrained the fully connected layer for these classification tasks.

Jnawali et al.^71^ used Inception-Resnet-V2 to detect thyroid cancer based on multi-spectral PA images and also applied transfer learning to train the model. They used a special device constituted by an acoustic lens and a rotary 1D detector array to perform C-scans. They used images at three wavelengths (760, 800, and 850 nm) as the input and achieved promising results. The area under the curve (AUC)^133^ was 0.73, 0.81, and 0.88 for cancer, benign nodules, and normal tissue, respectively, on *ex vivo* data. Then the authors stacked twenty-one 2D PA image groups taken at five different wavelengths to form a three-dimensional (3D) PA image cube, then used a seven-layer 3D CNN and an eleven-layer 3D CNN for cancer diagnosis.^73^ During training, the authors introduced a class-weight parameter to balance the distributions of positive and negative samples in the training data.^134^ The best 3D model (11-layer 3D CNN) could detect cancer with an AUC of 0.96 and the AUC of the 2D model was 0.72, which showed that 3D features could provide more information and that deeper models seemed to have stronger feature extraction capabilities. Based on this device, Dhengre et al.^75^ used several simple CNNs with the same architecture, or a combination of the CNNs with SVM or random forest (RF), to classify malignant, normal, and benign prostate hyperplasia prostate tissues (three binary classification tasks). The input of the network was a 105-timepoint A-line sequence in the region of interest. Involved models were: simple CNN, SVM, RF, SVM with features extracted by CNN (CNN_SVM), and RF with features extracted by CNN (CNN_RF). CNN_RF produced the highest sensitivity in most cases (highest to 0.993).

For mesoscopy imaging, Moustakidis et al.^74^ used traditional machine learning methods including ensemble learning methods and DL methods to identify skin layers in PA tomograms. These images were gotten by raster-scan optoacoustic mesoscopy (RSOM).^135^ In addition to applying simple CNN, they also used modified Alexnet and Resnet whose fully connected layer and classification layer were replaced by principal component analysis (PCA) and RF to classify 3D image directly. The simple CNN provided a classification accuracy of ∼85%, which was the best result in DL models. Nitkunanantharajah et al. used ResNet18^136^ to diagnose systemic sclerosis by identifying microvascular changes at the finger nailfold.^76^ The microvascular was also imaged by RSOM and segmented manually. The network’s input was the 2D images generated by frequency band equalization^137^ and maximum intensity projection. Transfer learning was applied to train the model, which ultimately achieved an AUC of 0.897, a sensitivity of 0.783, and a specificity of 0.895.

DL also played an important role in PA image segmentation, where U-Net is the most successful model. Chlis et al.^77^ applied an adapted sparse U-Net to perform vascular segmentation on clinical multi-spectral PA images. They used a 1×1 2D convolution layer to transform an image with 20 spectral channels (wavelengths from 700 to 970 nm in 10 nm steps) to a single-channel image. The model was trained on *in vivo* data acquired using a handheld PA/US system. The ground truth was established based on the consensus between two clinical experts. The performance of the sparse U-Net was similar to the standard U-Net whose size was 30 times bigger. They found that sparse U-Net was faster thus was more suitable for clinical application. Berkan et al.^78^ also showed the U-Net’s performance on segmentation of the mouse boundary in an (optoacoustic US) OPUS system. PA signal was detected by a 270-deg ring array and images were reconstructed by BP. They used manually segmented images as the ground truth. The dice coefficients on the cross-sectional images of the brain, liver, and kidney were 0.98, 0.96, and 0.97, respectively.

Boink et al.^68^ proposed a learned primal-dual (L-PD) model for simultaneous PA reconstruction and segmentation. Primal-dual algorithms are popular in tomography reconstruction.^138^^,^^139^ In their work, Boink et al. used a CNN-based model instead of the primal-dual hybrid gradient to learn the best update in each iteration during model-based image reconstruction. It consists of two networks: one for calculating the previous dual update and the other for calculating the primal update, which corresponds to the initial pressure. Compared to convex segmentation^140^ and U-Net, the L-PD method yielded the best results. There were two major innovations: (1) the data set was augmented by changing the number of detectors. (2) Deep-learning-based reconstruction was used to perform image segmentation. In other words, the authors reconstructed a segmentation map.

Motion artifacts and pixel dislocation are almost inevitable in optical resolution PAM of *in vivo* targets. These are attributable to the breathing motion and heartbeat of the animal, or to the positioning errors of the motor. Motion correction can improve the performance of image understanding. Chen et al.^79^ proposed a simple CNN with three convolutional layers to do motion correction. They employed it to process *in vivo* rat brain vessel images contaminated by motion artifacts. They found that the model’s performance was significantly improved using a larger kernel size, at the expense of longer processing time. Compared with several existing algorithms that are not based on DL,^141^^–^^144^ the proposed model demonstrated the best performance.

### 3.2 Reconstruction of Initial Pressure Images

**Fig. 3:**
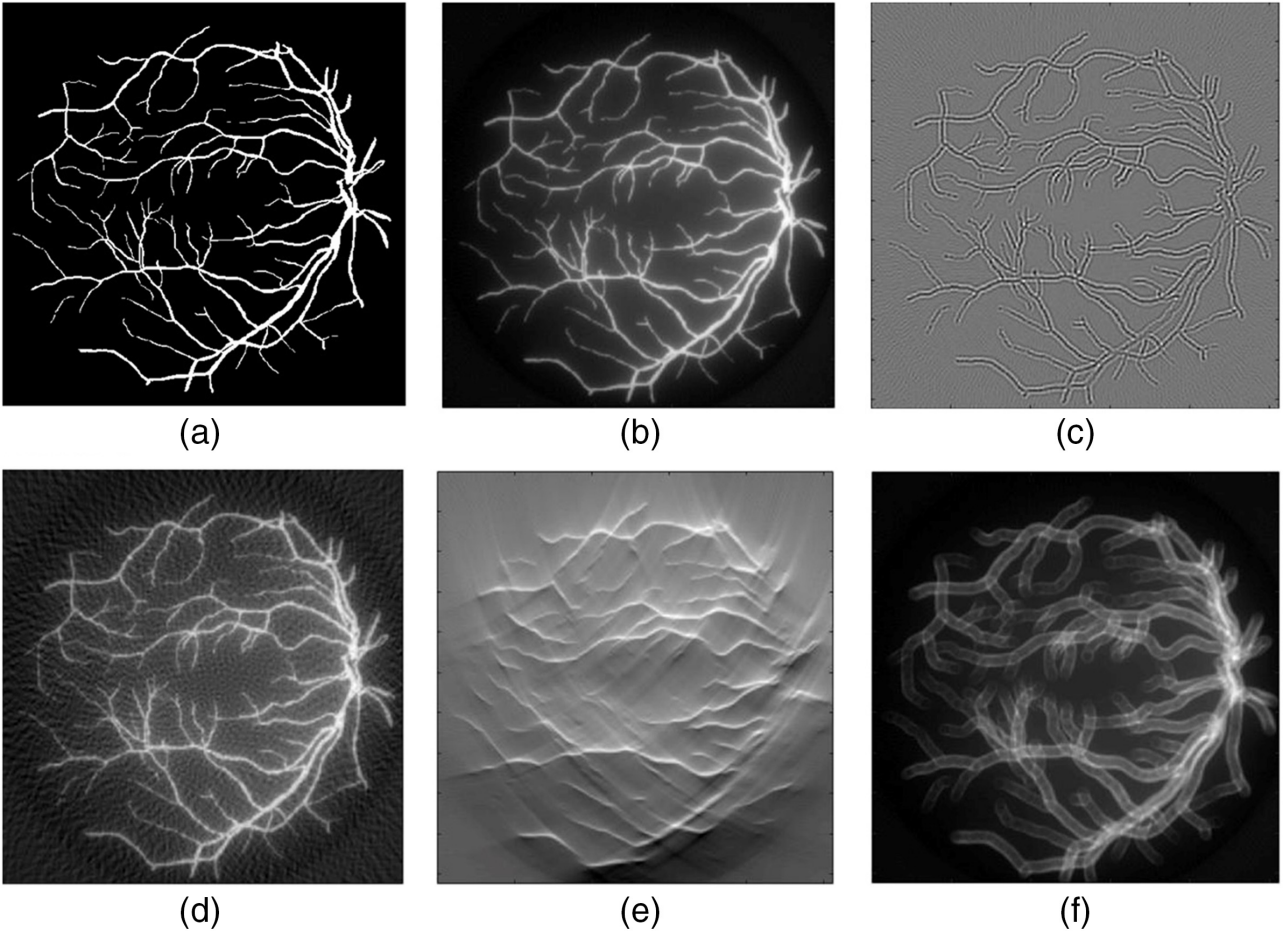
PA reconstruction quality is influenced by different non-ideal conditions: (a) gold standard; (b) good-quality reconstruction (DAS); reconstruction with reduced quality due to (c) limited bandwidth; (d) sparse sampling; (e) limited view; and (f) wrong SoS.

Traditional PA image reconstruction methods were introduced in Sec. 1. In brief, ideally a full detection solid angle of 4π, sufficient spatial sampling frequency, infinite detector bandwidth, and known distribution of the SoS, are the conditions for the traditional reconstruction methods to work properly. But these conditions are rarely satisfied in real applications—US transducers are non-ideal, and tissue properties can be complex and unknown—consequently, available information is inadequate for high-quality image reconstructions. The effects of information deficiency on the image quality are shown in Fig. 3, where the target was obtained from the DRIVE data set and simulated by k-wave.^145^ The applied reconstruction method is DAS. In some cases, signal quality is sacrificed for various purposes such as system cost. For example, LED are used to replace bulky lasers for system miniaturization.

DL methods were introduced to compensate for the above-mentioned signal deficiency. These methods can be classified into five types: (1) preprocessing of channel data: the NN is used to process the raw data, which is subsequently fed into a traditional reconstruction program to produce an image. (2) Postprocessing of reconstructed images: the network is applied to boost the quality of the images reconstructed by traditional methods. (3) Direct reconstruction of the initial pressure: the network directly outputs the initial pressure image when channel data are fed as the input. (4) Combining image reconstruction and postprocessing: the network improves image quality based upon both low-quality images and raw channel data. (5) Embedding DL in traditional reconstruction frameworks: use DL to perform certain calculations in traditional reconstruction methods (for example, to calculate the update or regularization term in model-based methods). Table 3 summarizes the network architecture for each kind of task and each type of model.

**Table 3:** Network architectures used in PA image reconstruction

Non-ideal condition		Preprocessing	Postprocessing	Direct reconstruction	Combined reconstruction	Embedded in traditional reconstruction
Single-non-ideal detection	Limited bandwidth	Simple NN^80^		U-Net^81^^,^^82^		
	Sparse sampling		U-Net^83^^–^^85^ and FD-Unet^86^^,^^87^ Simple CNN^89^ and complex CNN^90^			Simple CNN^88^
	Limited view		U-Net^91^^,^^92^ and VGG^92^	U-Net^91^ and complex CNN^93^	Multiple branches autoencoder^36^	
Non-uniform SoS			Faster R-CNN^94^^–^^97^ U-Net^99^^,^^100^	Simple CNN^98^		
Multiple non-ideal conditions		U-net^101^	U-Net;^32^^,^^37^^,^^102^ S-Net;^32^ WGAN (based on U-Net);^103^ and simple CNN^104^	U-Net^105^ and multiple branches autoencoder^106^	Ki-GAN (based on multiple branches autoencoder)^107^	U-Net;^108^^–^^110^ FD-Unet;^111^ simple CNN;^68^^,^^112^ multiple branches autoencoder; ^113^ and RNN^114^
Use of DL in economical and portable PAI devices	Single-channel DAQ			Autoencoder (based on LSTM)^115^		
	LED-PAI		Complex CNN^116^ and CNN&LSTM^117^	U-Net^118^^–^^122^		

The DL-based methods are often compared against traditional methods in terms of SSIM, PSNR, Pearson correlation coefficient (PCC), mean absolute error (MAE), MSE, normalized 2D cross-correlation (NCC), signal-to-noise ratio (SNR), contrast-to-noise ratio (CNR), and edge preserving index. These quantities were also added into the LF for better training.

#### 3.2.1 Single-non-ideal detection condition

Signal detections in PAI are often limited in bandwidth, sampling density, and angular coverage. For example, although linear arrays are commonly used in clinical settings, they suffer from all these limitations thus could only produce images with relatively low quality.^146^ In this section, we review how DL is applied to improve the image quality of PAI, where the role of DL is to compensate for the information loss due to one of the following: limited bandwidth, sparse sampling, and limited-view angle.

##### Limited bandwidth

Transducer bandwidth affects both the axial and lateral resolutions.^147^ Commercially available US detectors for medical imaging are typically narrowband, in contrast, PA signals are broadband, spanning from several tens of kilohertz to beyond a hundred megahertz.^20^ Although capacitive micromachined ultrasonic transducers and optical transducers may have larger bandwidth, they have not been widely adopted for PAI yet.^148^ A reduced bandwidth inevitably results in the loss of important image features.

In 2017, Gutte et al. used a five-layer fully connected deep NN to broaden the bandwidth of the channel data. The input and output of the network were both channel data (sinograms).^80^ The authors trained the network on numerical breast phantoms and tested its performance by simulated data (blood vessel network, Derenzo phantom, etc.) and real phantom data (horse hair phantom and ink tube phantom). Compared with least-squares deconvolution in terms of PCC, CNR, and SNR, the DL method almost tripled these measures in all the tests. Plus, it was almost 1.63 times faster than the least-squares deconvolution. In addition to deconvolving the frequency response, DL was also used to synthesize a broader frequency response given multi-band signals. Lan et al.^81^ used ConvU-Net, which was a modified U-Net with a kernel size of (20×3) and a stride of (20×1) as skipped connections, to reconstruct PA images from sinograms interlaced with signals individually acquired at three different center frequencies: 2.25, 5, and 7.5 MHz. It is a direct reconstruction model: the input of this network was the raw signal from 120 channels, containing three 40-channel subgroups that were distributed on a circle corresponding to the three center frequencies and the output was the reconstructed images. The authors trained and tested the network on numerical phantoms containing segmented vessels from fundus oculi CT imaging.^145^ ConvU-Net showed better image reconstruction quality than TR, DAS, and common U-Net in terms of SNR, PSNR, SSIM, and relative error. Lan et al.^82^ then used two U-Net structures connected end-to-end to reconstruct images from the three frequencies data. The model outperformed ConvU-Net in a series of tests.

##### Sparse sampling

The minimum spatial density to arrange detector elements is determined by the Nyquist sampling criterion. The problem in which the actual detector density is lower than the minimum required density is called sparse sampling. In many applications, spatial sampling is sparse, due to various reasons such as system cost and imaging speed. Visually, sparse sampling introduces streak artifacts and may jeopardize image resolution. Postprocessing models are often applied to address such problems.

In 2017, Antholzer et al.^83^ used a U-Net to process PA images that had been reconstructed by FBP based on sparse sampling data. The data were detected by 30 transducers evenly arranged around a circle. The authors trained their network on simulated data where Gaussian noise was added. Compared with the results obtained using FBP and the model-based method on simulation data, the DL method yielded the best quality. In addition, postprocessing by U-Net reconstruction required only 20 ms (FBP: 15 ms and U-Net: 5 ms) compared with 25 s for the model-based method. Guan et al. proposed a modified U-Net, termed FD-UNet, in which each layer is connected with every other layer. It was also a postprocessing model to remove streak artifacts. The original PA images were reconstructed by time reversal from sparse data.^86^ The training and testing data sets were generated from three different digital phantoms (circles, Shepp–Logan, and experimentally acquired micro-CT images of mouse brain vasculature) on different levels of sampling sparsity. FD-UNet performed obviously better than U-Net on PSNR and SSIM. When applied to the mouse brain vasculature data, FD-UNet outperformed U-Net by recovering more details. After transfer learning was applied, the networks’ performance was improved after fine-tuning with small and well-matched training data set. In 2020, Farnia et al.^84^ used a U-Net to process a PA image reconstructed using time reversal. Deng et al.^85^ used SE-Unet, a modified U-Net with added SE-blocks^149^ in skip connections, to remove artifacts in PA images reconstructed by BP. It got better results than BP and Sta-Unet.^91^

For PAM, DiSpirito et al.^87^ used FD-UNet to reconstruct images of *in vivo* mouse brain microvasculature blurred by under sampling. The fully sampled images were used as the ground truth and the images down-sampled at a ratio of 5:1 in one scan direction were employed as the input. Compared with U-Net, ResU-Net, and ResICL U-Net,^150^ FD-UNet had the best performance in PSNR and MSE and was the second best in SSIM. FD-UNet also showed good robustness, it did not require high image contrast, and performed stably well for images with different down-sampling ratios. Zhou et al.^90^ proposed a CNN-based model to improve the quality of PAM images suffering sparse sampling. Sixteen residual blocks and eight SE blocks were used for feature extraction after the first convolution layer of CNN. They claimed that the residual blocks performed well in super-resolution tasks and the SE blocks helped convergence. Compared with Bicubic interpolation and EDSR (another CNN-based method),^151^ the new method showed the best PSNR and SSIM on PAM images of leaf veins and *in vivo* data (blood vessels of the mouse eyes and ears).

Except U-Net, Awasthi et al.^89^ used a seven-layer network to achieve super-resolution and denoising on the channel data. The training dataset was simulated by the CHASE,^152^ DRIVE,^145^ and STARE^153^ databases. Their simulation involved 100 detectors, and Gaussian noise (SNR to 20/40/60  dB) was added. They then spatially down-sampled the data by a factor of 2, resulting in a reduced channel number of 50. Finally, the down-sampled data was interpolated to recover a total channel number of 100 as the input. The output of the network was the residual between the interpolation result and the initial signal detected by all the detectors. On average, the network reduced RMSE by 41.70% while increased PSNR by 6.93 dB on simulated data (numerical blood vessel and Derenzo phantoms) and *in vivo* data (rat brain).

Image reconstruction incorporating DL and model-based methods have been also explored. Antholzer et al.^88^ developed a simple NN consisting of three convolutional layers to calculate the regularization term in model-based image reconstruction, termed network Tikhonov (NETT). They built a training set of input/output pairs, in which the inputs were images reconstructed by FBP from under sampling data and the outputs were the differences between the predicted signal and the ground truth to train the regularization. When the input was the ground truth, the output was zero. Compared with FBP, a compressed sensing method with $l_{1}$-minimization,^154^ a model-based method with $H^{1}$-regularization, and U-Net (used as a postprocessing method), NETT produced the best results with noise-free data and satisfactory results with noisy data.

In this section, we show how DL can remove artifacts generated by sparse sampling. Similar approaches were reported by Schwab et al.^155^ and Guan et al.^111^ Since these works deal with situations beyond merely sparse sampling, they will be discussed in Sec. 3.2.3.

##### Limited-view detection

Incomplete angular coverage can distort image features by partially losing information and generating artifacts. The problem is frequently encountered in PAI and is termed “limited view.” Starting from 2018, DL has been extensively studied to solve the limited-view problem. Because in the missing cone signal is completely lost, tackling limited view is more of a super-resolution than a deconvolution problem.

In 2018, Waibel et al. investigated both postprocessing and direct reconstruction to solve the limited-view problem.^91^ They used a U-Net to do postprocessing, and a modified U-Net with an additional convolutional layer that resized the information to the target resolution in skip connections, to reconstruct PA image directly. In their simulation, the PA signals generated by circular and elliptical targets were detected by a linear array. The median relative estimation error improved from 98% (DAS) to 10% (postprocessing) and 14% (direct reconstruction). The direct reconstruction method was faster than postprocessing because it did not need the DAS step. Deng et al. proposed a postprocessing method, in which they built an *in vivo* data set and used two DL models to improve *in vivo* image quality. They utilized the PA signal acquired by a ring array to generate the data set.^92^ The PA images reconstructed from a full ring were used as the ground truth, and images reconstructed from a partial ring (1/4 ring, 90-deg coverage) were used as the images with limited-view artifacts. (A similar approach was demonstrated by Davoudi et al.^37^ and will be discussed in Sec. 3.2.5.) They used U-Net to postprocess the images reconstructed by DAS. They also mapped the full-view images, all taken at the liver region of the mouse, into a multi-dimensional space (20 in their work) and calculated the bases and their weights by PCA. VGG was then used to establish the mapping between the projection weights of the full-view and the limited-view images. These models all produced satisfactory results.

For direct reconstruction, Anas et al.^93^ proposed a model on the basis of CNN. They used dilated and large convolution kernels at higher layers to obtain global information to prevent resolution loss. They performed simulations (using circular targets) to train, validate, and test the network. This model could obtain a nearly 8.3 dB increase of PSNR. Lan et al. proposed a hybrid autoencoder model, called Y-Net. It consists of two intersecting encoder paths that got information from reconstructed images or raw data to solve the limited-view problem in linear array PAI systems. The authors compared the performance of Y-Net with that of time reversal, DAS, and U-Net (postprocessing) in terms of SSIM, PSNR, and SNR on simulated data and experimental data (chicken breast tissue inserted with two pencil leads and a *in vivo* human palm), Y-Net got the best results. Moreover, Y-Net achieved a better “transfer” ability than U-Net because it referred to information in the raw data.

There are works that addressed both the sparse sampling and limited-view issues as will be introduced in Sec. 3.2.3.

#### 3.2.2 Non-uniform speed of sound

The SoS in biological tissues is generally non-uniform, with unknown spatial distributions. In addition, a SoS mismatch often exists between the biological tissue and its surrounding medium. In all cases, if instead a constant SoS is assumed during the image reconstruction process, artifacts will be generated (such as feature splits). Moreover, a large SoS discontinuity (e.g., between muscle and bone) is bound to create acoustic reflections. The reflected signals can mix with the PA signal to introduce reflection artifacts. Recent studies have shown the great potential of DL in solving the acoustic heterogeneity problem in PAI. Researchers first treated non-uniform SoS as a target identification problem, addressing whether an image feature is real or fake. Other researchers used DL to reconstruct images directly or to perform postprocessing on PA images originally generated using traditional methods.

In 2017, Reiter and Bell^98^ trained an eight-layer simple CNN to predict realistic images (point targets) in heterogeneous media directly from raw data. The model achieved satisfying results (mean axial and lateral point location errors of 0.28 and 0.37 mm, respectively) at various sound speeds (1440 to 1640  m/s), target locations (5 to 25 mm), and absorber sizes (1 to 5 mm) using simulated data. This work predicted the point locations successfully but failed to identify whether the wave front was from the actual target or the reflection of the target. Allman et al. implemented a series of faster R-CNN models based on VGG, Resnet-50, and Resnet-101 to decide whether the identified features were actual targets or reflection artifacts in PA images reconstructed by DAS.^94^^–^^97^ Faster R-CNN is an object detection framework based on deep convolutional networks. It can provide bounding boxes and confidence scores for target detection. In their works, faster R-CNN output the type of the objects (i.e., source or artifact), along with their locations in the form of the coordinates of the bounding box and a confidence score between 0 and 1 for each object. The authors trained their models on simulated data and tested them using phantom data, *in vivo* data (an optical fiber in a pig vessel) and *ex vivo* data (a needle tip inserted into tissue). PA signals were detected using a linear array. Faster R-CNN was able to accurately identify and locate the targets. All three DL models produced satisfactory results on phantom data and *ex vivo* data. For *in vivo* data, the residual network architectures correctly classified 83.3% (Resnet-50) and 88.8% (Resnet-101) of the sources. The authors suspected that a deeper network (i.e., residual network) has a greater capacity to learn higher-level features.

Other than point targets, DL was also studied to deal with acoustic heterogeneity for general targets and these are all postprocessing models. In 2019, Shan et al.^99^ used a modified U-Net for postprocessing images after the first iteration of a model-based algorithm. The input of the U-Net was the feature map extracted from the PA images by a four-layer CNN, whereas the output of the U-Net was processed by a network consisting of four deconvolution layers as the final reconstructed image. They added image structure information (SSIM) in the LF. The authors trained the network and tested its performance using data generated based on cadaver CT scans. In their simulations, they randomly picked a circular region where SoS was set to be high and introduced a sinusoidal change of SoS in the background tissue. And 0%, 10%, 20%, 30%, and 40% noise was added to the PA signals. The DL method produced the best results compared to that of TR and other popular iterative algorithms, such as averaged time reversal (ATR),^156^ adjoint ATR,^157^ and Landweber iteration.^158^ In addition, without doing iterations, the DL method was the fastest among all compared algorithms. To compensate for the SoS heterogeneity, Jeon and Kim^100^ proposed an autoencoder model whose architecture was similar to U-Net. The data were also contaminated by noise and beamformed with various SoS to generate the multi-channeled input data (eight channels with eight speeds). The ground truth image was obtained by reconstructing the noise-free data with the correct SoS. The model was able to remove image artifacts associated with acoustic heterogeneity, even though it had never been trained with the correct SoS map on *in vivo* data (forearm). In addition, the model was also effective in reducing side lobes and noise.

#### 3.2.3 Multiple non-ideal conditions

In Secs. 3.2.1 and 3.2.2, we have shown that DL can be used in applications where a single-non-ideal detection condition dominates. In real cases, often times the combination of several such conditions may conspire to reduce the image quality. We have shown that certain DL networks, such as U-Net and its variants, were successfully applied to deal with individual factors including sparse sampling, limited view, and acoustic heterogeneity. Consequently, such networks are expected to be applicable when several of these factors simultaneously take effect. Here we introduce DL methods that work in conditions where multiple non-ideal factors co-exist. These techniques are more promising for practical uses.

In this section, we will first introduce the preprocessing and postprocessing models. Second, direct reconstruction models will be discussed. Third, we will introduce the combined models that use raw data and reconstructed image as inputs. Finally, we will introduce how DL can be used in conjunction with traditional reconstruction methods, for example, DL networks can be used to calculate the sum in DAS or compute the update in model-based methods.

For preprocessing models, Awasthi et al.^101^ proposed a modified U-Net for super-resolution and bandwidth extension. Named U-Net (hybrid) and directly working on the channel data, the proposed network used exponential linear units (ELUs) as the activation function in the final layers and used ReLUs in other layers.^61^ The input of the network was the signal detected by 100 bandwidth-limited detectors, and then the data were interpolated to 200 channels employing the nearest neighbor method. The ground truth data were generated with 200 full-bandwidth detectors. They divided the channel data into small subsections, the size of each subsection was 64×64. The network only processed one such subsection at one time. Compared with SRCNN, U-Nets (implementing only ReLU or ELU), direct interpolation, and automated wavelet denoising,^159^ the proposed network got the highest SNR for phantom data (horse hair) and *in vivo* data (rat brain).

**Fig. 4:**
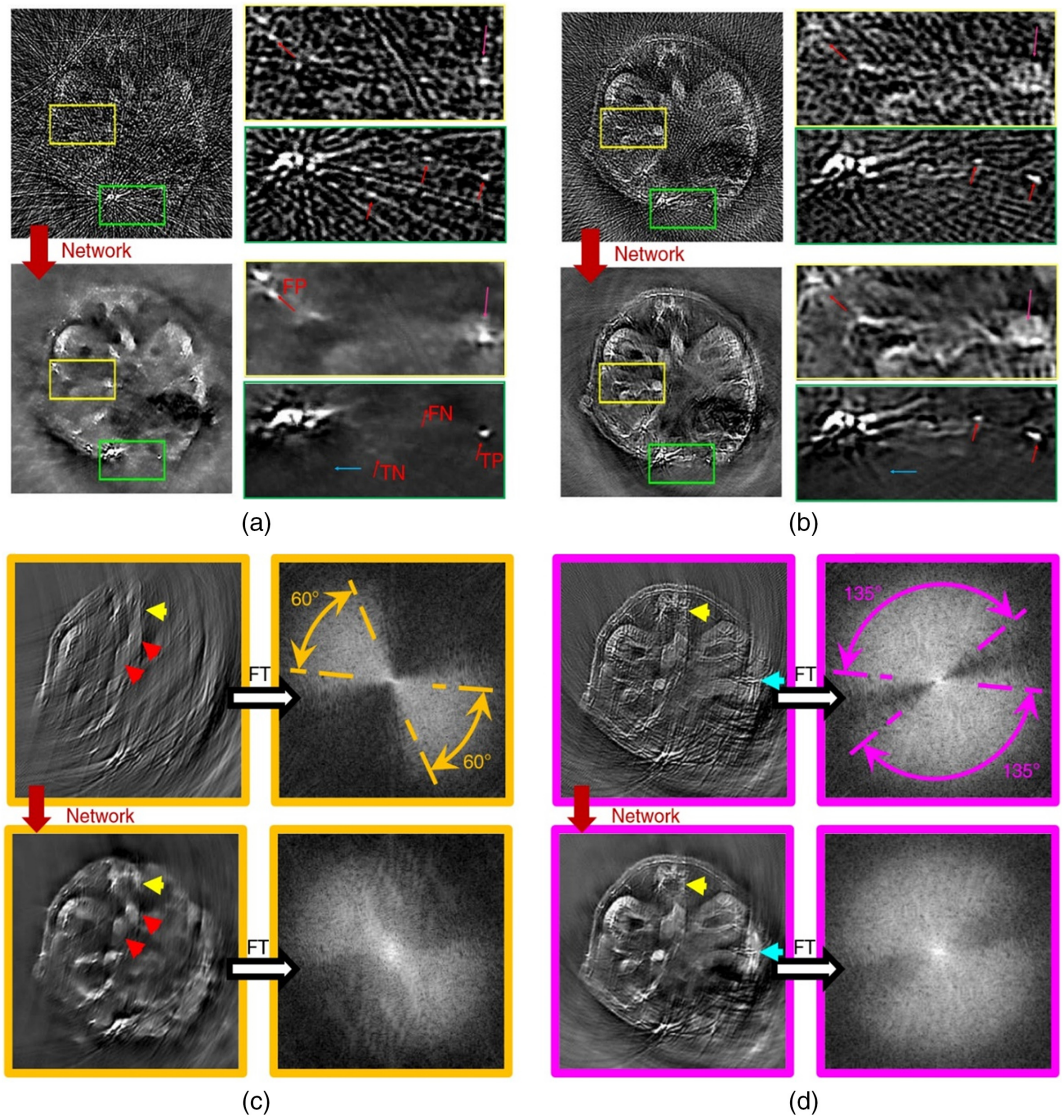
The results of Davoudi et al.’s method. (a) Reconstructed image with under sampled (32 projections) data versus its artifact-free counterpart obtained with the trained network. TP, true positive; FP, false positive; TN, true negative; and FN, false negative. (b) Another example for under sampled data with 128 projections. (c) Top: image reconstructed with 60-deg angular coverage and its respective amplitude spectrum. Bottom: output image of the network and its corresponding amplitude spectrum. (d) Top: image reconstructed with 135-deg angular coverage and its respective amplitude spectrum. Bottom: output image of the network and its corresponding amplitude spectrum.

In an attempt to apply DL to the postprocessing of PA images, Antholzer et al.^32^ used a U-Net and an S-Net (simple network consisting of three layers) to enhance PA images originally reconstructed by FBP in limited-view and sparse-sampling conditions. In the numerical study, 24 sensors were located on a non-closed curve (less than a semicircle). Both networks were successful in removing most of the artifacts. The more complex U-Net produced better results but also took a longer time to train and process the images. Davoudi et al.^37^ also employed U-Net to perform postprocessing on sparse sampling and limited-view images. They obtained a full-view image from a full-view tomographic scanner with 512 transducers in a circle as the ground truth. Sparse-sampling and limited-view data were gotten by down sampling. The training data set was constructed using images of 6 mice with 100 different cross sections from each. Their model produced satisfying results on *in vivo* data, as shown in Fig. 4. The authors also made their data set available online.

Godefroy et al.^102^ introduced the Bayesian machine learning framework into PA image postprocessing Bayesian machine learning framework into PA. They applied MC dropout as a Bayesian approximation in U-Net.^160^ So the model was able to generate 20 different outputs for the same input. The input was the images reconstructed by DAS, and the prediction image was the mean of the different outputs. In their experiment, PA images taken by a linear array were used as the input, and the photographs taken by a CMOS camera were used as the ground truth. Test results showed that the model effectively improved NCC and sSSIM and was more robust than networks without dropout.

Vu et al.^103^ used Wasserstein generative adversarial network with gradient penalty (WGAN-GP) to do postprocessing on PA images to remove artifacts due to limited-view and limited-bandwidth detection. The generator in WGAN-GP took the form of a U-Net, and the discriminator of this model was a common CNN. The discriminator was trained to identify the ground truth from the generator’s output. The generator’s input was the PA image reconstructed by TR. They used a complex LF to train the network. It was the sum of three factors: (1) the distance between the discriminator’s output and the true distribution. The Wasserstein distance was employed here to take advantage of its capability in describing morphological characteristics. (2) The GP, which could avoid gradient explosion or disappearance. (3) The MSE loss, which helped preserve the information in the reconstructed images. In simulation, phantom, and *in vivo* experiments, WGAN-GP all showed slightly better performance on SSIM, PSNR, and in recovering low-contrast structures than U-Net.

Zhang et al.^104^ developed a nine-layer CNN to solve the under-sampling and limited-view problems on PA images reconstructed by UBP. The PA signal was detected by a three-quarter ring transducer array with 128 elements. The network was trained on simulated 3D vascular patterns. They also trained and tested a compressed sensing model and in all numerical, phantom, and *in vivo* experiments, the CNN got better results.

For direct reconstruction, in 2020, Feng et al.^105^ used a Res-UNet network to reconstruct PA images. In their work, signals were detected by 128 transducers covering an angle of 270 deg around the imaged object. They added a residual connection on the layer of a U-Net and a convolution layer was introduced as a copy-and-crop path in the network. Generalization of the ResU-Net network was achieved by imaging six types of synthetic phantoms, including discs, breads, spiders, lines, logos, and natural pictures. They compared the network’s performance with U-Net on digital and physical phantoms. Res-UNet got the best PC and PSNR on digital phantom and the images with the least artifacts on physical phantom. Inspired by the fact that FBP takes the time derivative of the signal as input, Tong et al.^106^ proposed a feature projection network (FPnet) to reconstruct PA images based on limited-view and sparsely sampled data. Its architecture resembled that of Y-net with the two input branches fed with the channel data and the time derivative of the channel data, whereas the output was the initial pressure image. Then they used U-Net to do postprocessing on the image. The outputs of FPnet and U-Net were trained on simulated and *in vivo* data to match the ground truth. The hybrid model showed better results compared with: (1) FPnet only, (2) postprocessing (by U-Net) images reconstructed by FBP, and (3) other traditional methods (FBP and model-based).

**Fig. 5:**
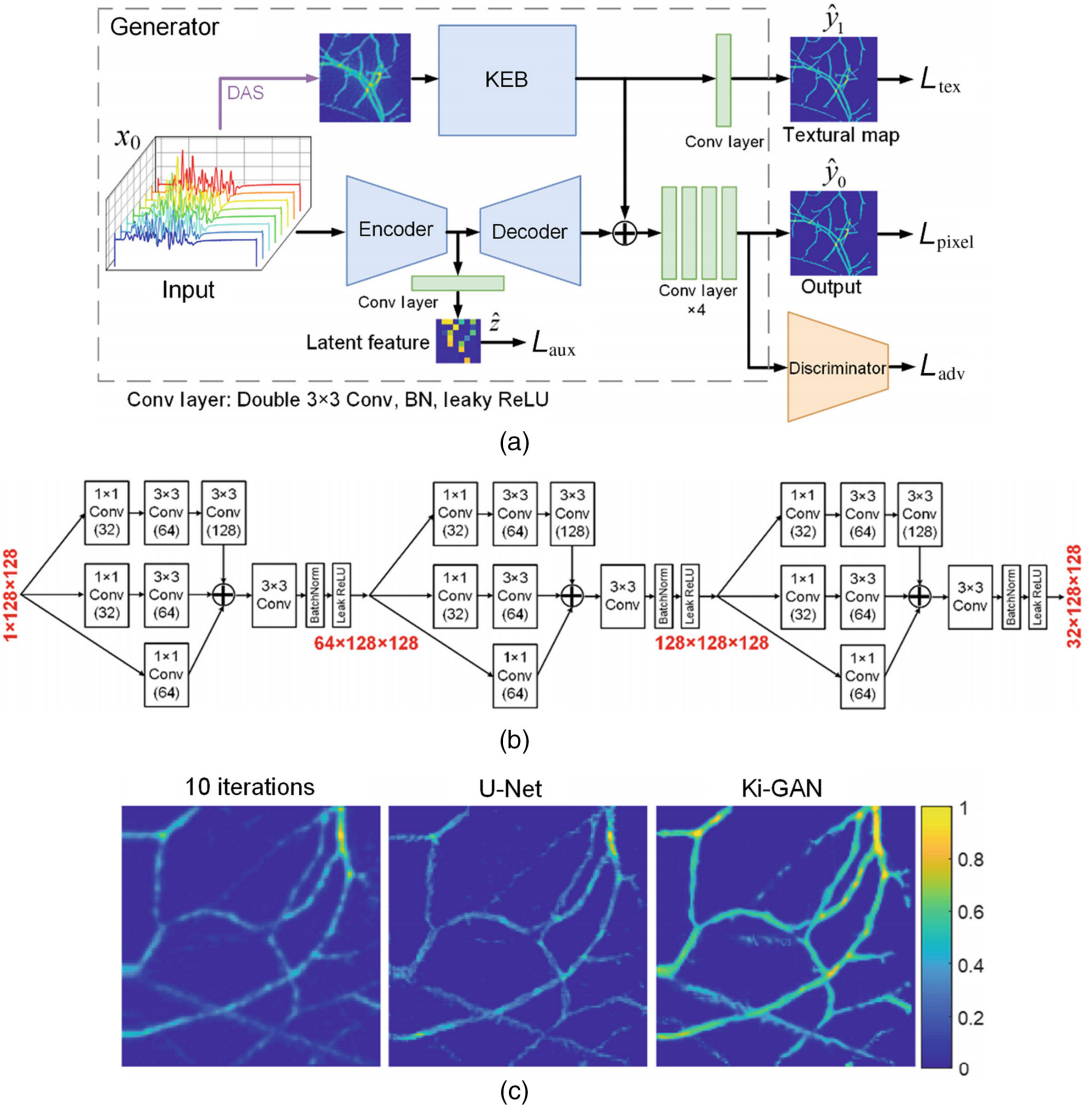
The architecture of Lan et al.’s model. (a) The overall architecture of Ki-GAN; KEB represents convolutional layers; DAS: delay and sum reconstruction. (b) The detailed architecture of KEB. (c) PA images of rat thigh reconstructed by iterative algorithm with 10 iterations (column 1), U-Net (column 2), and Ki-GAN (column 3).

Lan et al. proposed a knowledge infusion generative adversarial network (Ki-GAN). As a combined network, Ki-GAN had two branches, which got information from reconstructed images and the raw data.^107^ Compared with Y-net, Ki-GAN had a more complex architecture. A knowledge embedding branch (KEB, which is a CNN with a three-layer inception block architecture) was used to extract information in the generator. Information from the two branches were merged by a four-layer CNN to reconstruct the final image. The architectures of Ki-GAN and KEB are shown in Figs. 5(a) and 5(b). The discriminator penalized the texture at the scale of patches.^161^ The model implemented an information-rich LF in the form of the linear combination of several MSE loss terms to improve feature extraction. Compared to Ki-GAN with DAS, U-Net (including direct reconstruction and postprocessing), and the iteration method (10 steps), Ki-GAN yielded the best results in terms of SSIM, PSNR, and SNR. The results of the *in vivo* experiment are shown in Fig. 5(c). Meanwhile, Ki-GAN was much faster than the iteration method (0.025 versus 331.51 s).

In traditional PA image reconstruction, certain computation tasks can be accomplished using DL for stronger fitting capability and faster speed. For example, DL can be applied to learn the parameters of traditional reconstruction methods. Dynamic aperture length (DAL) correction is one solution to the non-ideal detection problem.^162^^,^^163^ In 2018, Schwab et al. proposed a framework named DALnet, in which an image was first reconstructed by UBP with DAL correction, and then processed by a CNN network (U-Net with a residual connection) to address the limited-view and under-sampling problem.^108^ The weights of the DAL correction and the U-Net were jointly trained. The output of the model was compared with those of UBP and a model-based method in terms of total variation, and DALnet was superior in both image quality and computing speed. After this work, Schwab et al. also proposed a similar network to learn the weights in UBP to improve the PAT image quality under limited-view and sparse-sampling conditions.^155^ The network only had two layers. The first layer received raw data as input and carried out temporal filtering without any trainable weights, and the second layer performed back projection with adjustable weights. The following conditions were considered: limited view, sparse sampling, and limited-view plus sparse sampling. The network-assisted UBP reduced the relative errors of conventional UBP from 0.2002, 0.3461, and 0.3545 to 0.0912, 0.1806, and 0.1649, in the three conditions, respectively.

Other approaches of combining FBP/DAS with DL have been developed as well. Under the non-ideal detection conditions, DL has been used in FBP/DAS to replace the sum operation to restore some of the lost information. Guan et al.^111^ proposed a DL model called pixel-wise DL (Pixel-DL) for image reconstruction under limited-view and sparse sampling conditions. In Pixel-DL, they back-projected the interpolated channel data to the image grid using a constant SoS. Then the projected data group consisted of multiple channels were put into FD-UNet for image reconstruction. The authors compared the performance of Pixel-DL to those of other PAT image reconstruction methods, including TR, iterative reconstruction, post-DL (using FD-UNet to process an image reconstructed by TR), and modified Direct-DL (using FD-UNet to reconstruct PA image from raw data). Pixel-DL generated comparative results with the iterative method, and both were better than the rest. In 2019, Kim et al.^109^ employed the same concept and proposed a model using U-Net to reconstruct images in a linear array setting. The authors reformatted the detected PA signals into multi-channel 3D (tensorial) data using prior knowledge about the acoustic propagation delays and utilized a U-Net model to produce reconstructed images. Compared with DAS, DMAS,^164^^,^^165^ model-based, and U-Net (for postprocessing), the new model produced the best results when applied to simulated data. For the phantom and *in vivo* data, the model provided superior contrast and resolution.

The model-based methods can be used to reconstruct images in the non-ideal detection conditions. In some cases, high-quality images can be produced and used as the ground truth.^108^ However, implementation of model-based reconstructions is often iterative, making them time-consuming; to accelerate their speeds, DL models can be used.

Hauptmann et al.^112^ proposed a deep NN that learned the entire iteration process of a model-based reconstruction algorithm for limited view and sparse sampling. The method was called deep gradient descent (DGD). The current result and the gradient of the LF were used to calculate the output of the next iteration by using a five-layer CNN. The network used a greedy approach, in which every network output was used to match the ground truth. The planar array used in the study inherently had a limited view, and sparse sampling was performed by a 16× random selection of transducer elements. When applied to simulated data, after two iterations DGD was able to match the image quality of the traditional iterative method after 50 iterations. Transfer learning was used to advance the training for the *in vivo* data. They used fully sampled images reconstructed by the model-based method as the gold standard. The DGD after retraining produced satisfactory results. In addition, the iterative methods seemed to be more robust than U-Net, which was more sensitive to data variations. Later, Hauptmann et al.^110^ proposed a modified DGD, called fast-forward PAT, which employed a multi-scale network, like a shallow U-Net, to update the results. According to the test, with 4× subsampled data, FF-PAI with five iterations produced comparable results to the model-based method with 20 iterations. Compared with the conventional model-based method, FF-PAI reduced the computation time by a factor of 32. Boink et al.^68^ proposed an L-PD model to perform image reconstruction and segmentation simultaneously using the same network, and their method has been introduced in Sec. 3.1. The result of image reconstruction and segmentation was generated in the first and second channel, respectively. According to the authors, L-PD produced better results than FBP, model-based method with TV, and postprocessing with U-Net, when noise and sparse sampling were concerned.

Shan et al.^113^ developed a CNN-based model to reconstruct the SoS and initial pressure jointly using the framework of the model-based method. The updates of initial pressure and SoS were computed based on three types of input: (1) the initial pressure distribution, (2) the negative gradient of the L2 distance between the reconstructed image and the ground truth, and (3) the current SoS distribution. Feature extraction, feature fusion, and image reconstruction were all processed by the CNN. The model produced satisfactory results, where the MAE for SoS and the initial pressure were, respectively, $(0.85\pm 0.33)\times 10^{-2}\;\mathrm{Pa}$ and 1.24±0.52  m/s (mean ± std) after four iterations on simulation data.

Yang et al.^114^ proposed a recurrent inference machine (RIM) based on an RNN to calculate the update in each iteration step. The reconstruction results and the gradient of the objective function (optimization equation) were used as inputs for the next iteration step. Gated recurrent unit was used in this network.^166^ This unit has an update gate and a reset gate to avoid information loss. The first iteration input was calculated by multiplying the detected signal by the adjoint of the acoustic transmission matrix. The authors compared their results with those obtained using the DGD algorithm with five iterations, and RIM produced slightly better result. Compared with U-Net, RIM reduced the number of parameters by almost fourteen times.

#### 3.2.4 Use of DL in economical and portable PAI devices

DL was applied in some PAI systems to help achieve device miniaturization, cost reduction, and image quality improvement. We will first introduce how DL helps to reconstruct images based on single-channel DAQ. Then we will show how DL is used to enhance image quality in situations where the light intensity is low (e.g., when LED are used), and the role of DL can be seen as performing segmentation between target and noise. Again, U-Net and its variants are the most popular architectures used.

In 2019, Lan et al.^115^ developed a PACT system to provide real-time imaging. The imaging system consisted of 120 sensing elements connected to a DAQ unit with only a single channel. In their system, the 120-channel signals were overlaid directly into 4-channel signals before being fed into a delay-line module, allowing the signals to be temporally separable and combined into a single channel. Subsequently, the final output (single channel) could be separated into four channels, from which the authors used a DL network to reconstruct the PA images. The architecture of the network was also an autoencoder where the encoder was LSTM and the decoder was CNN. They were connected by fully connected layers. Compared to DAS with 120 channels, the proposed model could perform image reconstruction faster (28 versus 159 ms) with satisfactory result on phantom data (four black balls in agarose gel). This demonstrated the potential of the single-channel DAQ for high-speed low-cost operation.

Pulsed laser diodes (PLD) and LED are stable, affordable, and compact alternative light sources for PAI. However, their output energy is low, typically in the range of nJ/pulse to μJ/pulse.^118^ The low energy leads to low signal intensity and thus reduced image quality. A favorable property of PLD/LED is that they have high repetition frequency, typically on the order of kHz and above. A common method for denoising is averaging, at the expense of temporal resolution. Yet, even with averaging the image quality of the PLD/LED-based systems is suboptimum compared to their bulk-laser-based counterparts.

**Fig. 6:**
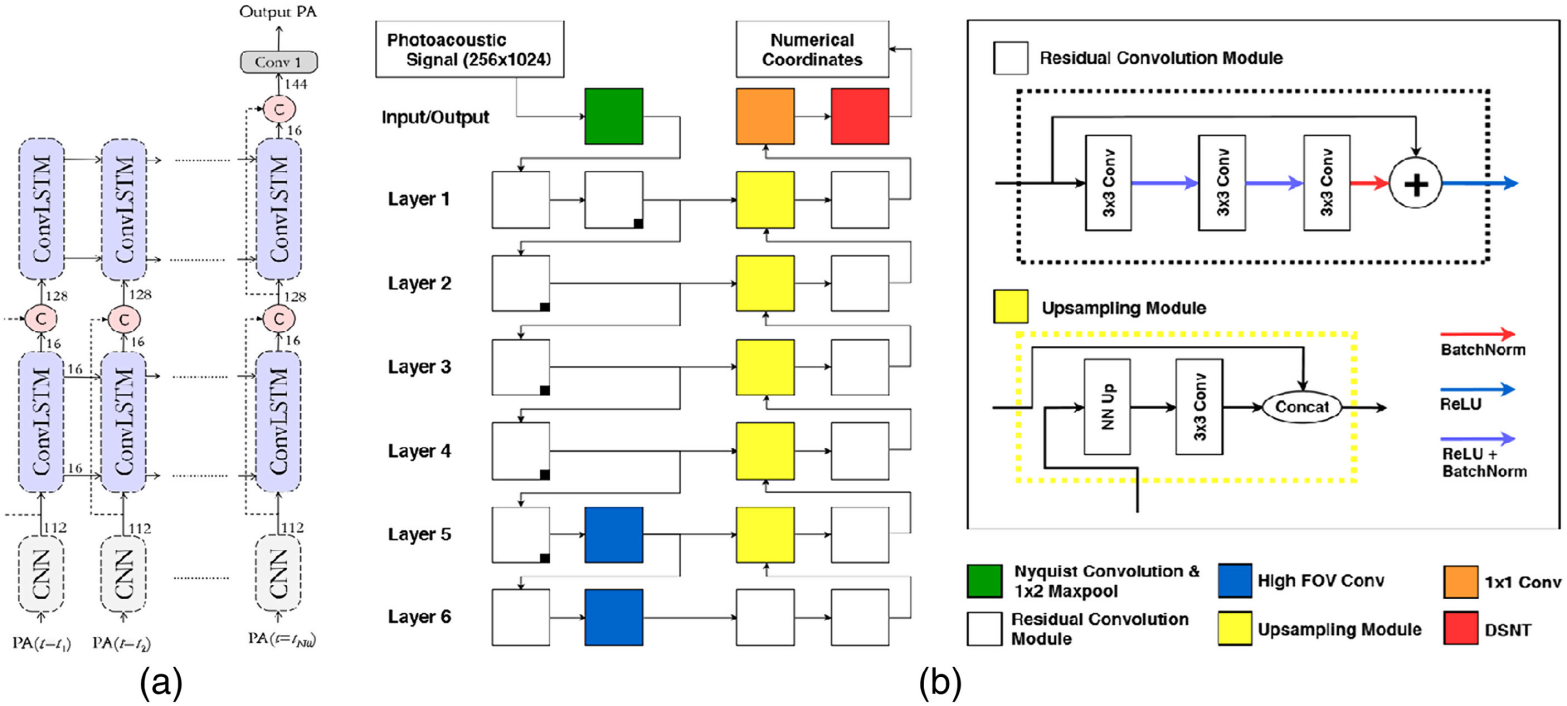
The architecture of (a) Anas et al.’s network and (b) Johnstonbaugh et al.’s network. Details of the residual convolution module and the upsampling module are also provided.

In 2018, Anas et al. proposed a model that consisted of series of dense convolutional layers^167^ to improve the quality of a 2D LED-based PA image.^116^ In addition to the final output, it also has two outputs in the middle layers that were all fed into LF to train model. A number of raw data frames were averaged and reconstructed by DAS as input. The model could increase frame rate by 6 times compared to the conventional averaging approach, with a mean PSNR of 34.5 dB and a mean SSIM of 0.86. Then Anas et al.^117^ combined CNN and ConvLSTM to improve the quality of these images. Its architecture is shown in Fig. 6(a). The inputs of the network were a sequence of PA images. The model used CNN to extract spatial features, whereas ConvLSTM^168^ was employed to exploit the temporal correlation among the images. The input is a sequence of averaged PA images. In a phantom experiment (phantom: a wire and a tube filled with gold nanoparticles), the network increased the frame rate by 8.1 times compared to the CNN-only method.

Kerrick et al.^119^ also proposed an encoder–decoder with atrous convolution (called as Nyquist convolution) on input layer to predict the location of circular chromophore targets in tissue mimicking a strong scattering background. It can achieve micron level accuracy on simulated data. For *in vivo* data, the network significantly improved the image quality. In 2019, Johnstonbaugh et al.^120^ also proposed an encoder–decoder model for the direct reconstruction of point targets under low illumination intensity. The model was based on U-Net whose architecture is shown in Fig. 6(b). In each of the last two layers, a high field-of-view convolution module (5×5) was applied in between the encoder and decoder. The down sampling designs (Nyquist convolution) reduced the network size and sped up the calculations. The model successfully imaged a deep vessel target with an accurate estimation of its position on AcousticX system, while the corresponding image reconstructed by FBP was noisy and difficult to interpret. The model also produced good multi-target localization results.

For postprocessing tasks, U-Net was typically used. Hariri et al. used a modified U-Net, termed multi-level wavelet-CNN, in which the pooling operations were replaced by discrete wavelet transform (DWT), and pooling operations were replaced by DWT, and the upsampling operations were replaced by inverse wavelet transform, to enhance PA image quality in a low SNR setting.^118^ They used the PA images taken at a fluence of 17  mJ/pulse as the ground truth and then reduced the laser fluence down to 0.95 and 0.25  mJ/pulse to train the network. In *in vivo* experiment, mice injected with various concentrations of methylene blue (MB) were imaged, and the CNR improvement factor was 1.55, 1.76, 1.62, and 1.48 for a dye concentration of 0.05, 0.1, 1.0, and 5.0 mM, respectively. Similarly, Singh et al. used a U-Net network to enhance the image quality of LED-based PAI.^121^ The targets were small tubes filled with MB or indocyanine green (ICG) in a water bath. PA images were obtained using one LED system and two OPO systems. The network was trained with images obtained by the two OPO systems and tested with images acquired by the LED-based system. The DL network improved the SNR by ∼30%. Manwar et al.^122^ also reported a similar idea and used U-Net to enhance the SNR of deep structures in brain tissue. They got B-scan images from an *ex vivo* sheep brain by a linear array at 20 and 100 mJ as the input and the label, respectively. They also evaluated several LFs including MAE, MSE, SSIM, multi-scale SSIM (MS-SSIM), and some combinations of these factors (MS-SSIM + MSE and MS-SSIM + L1). The network can enhance image quantity effectively, and the networks with combined LF got better performance. To improve SNR, some novel network structures can be used, such as denoising convolutional neural network.^169^

### 3.3 Quantitative Photoacoustic Imaging

With known spectral profiles of the major tissue chromophores, the goal of QPAI is to image the concentrations of the molecular targets through a process known as the optical inversion. Because of wavelength-dependent light absorption and scattering, the fluence spectrum in deep tissue is unknown, thus the absorption spectrum cannot be directly inferred from the PA spectrum. Traditional QPAI strategies often rely on overly ideal assumptions, such as piecewise constant optical properties, *a priori* knowledge of scattering coefficients, and homogeneous (and known) background optical properties.^22^^,^^35^ Among the developed methods, model-based iterative optimization can provide relatively accurate solutions but are time-consuming and sensitive to quantification errors in the PA images.^170^^,^^171^ Diffuse optical tomography can facilitate fluence distribution estimation but tends to increase system complexity and cost.^172^ In recent years, DL methods were applied to solve the QPAI problem with great success. The main reason why NNs are suitable for QPAI is that they are good at solving non-linear problems. Autoencoder with the end-to-end architecture is suitable for QPAI, in which the input is the PA images acquired at different wavelengths, whereas the output is the concentration map of the target molecules. In these tasks, U-Net and its variants as well as fully connected networks were commonly applied. Most of the works involved estimating the concentration maps for the whole tissue or manually segmented regions of interest. However, the prediction results were poor in areas with weak optical absorption. It would be beneficial to extract trusted regions with sufficiently high SNR for quantitative analysis. The DL-assisted QPAI tasks and applied networks are shown in Table 4.

**Table 4:** Network architectures used in QPAI

Task categories	Network architecture
Calculate ${\mathrm{sO}}_{2}$, chromophore concentration, or fluence spectra	U-Net,^35^^,^^123^^–^^129^ Autoencoder,^130^ Simple NN,^131^ and LSTM&CNN applied in traditional methods^132^
Generate mask for trusted zones	U-Net^127^^–^^129^

**Fig. 7:**
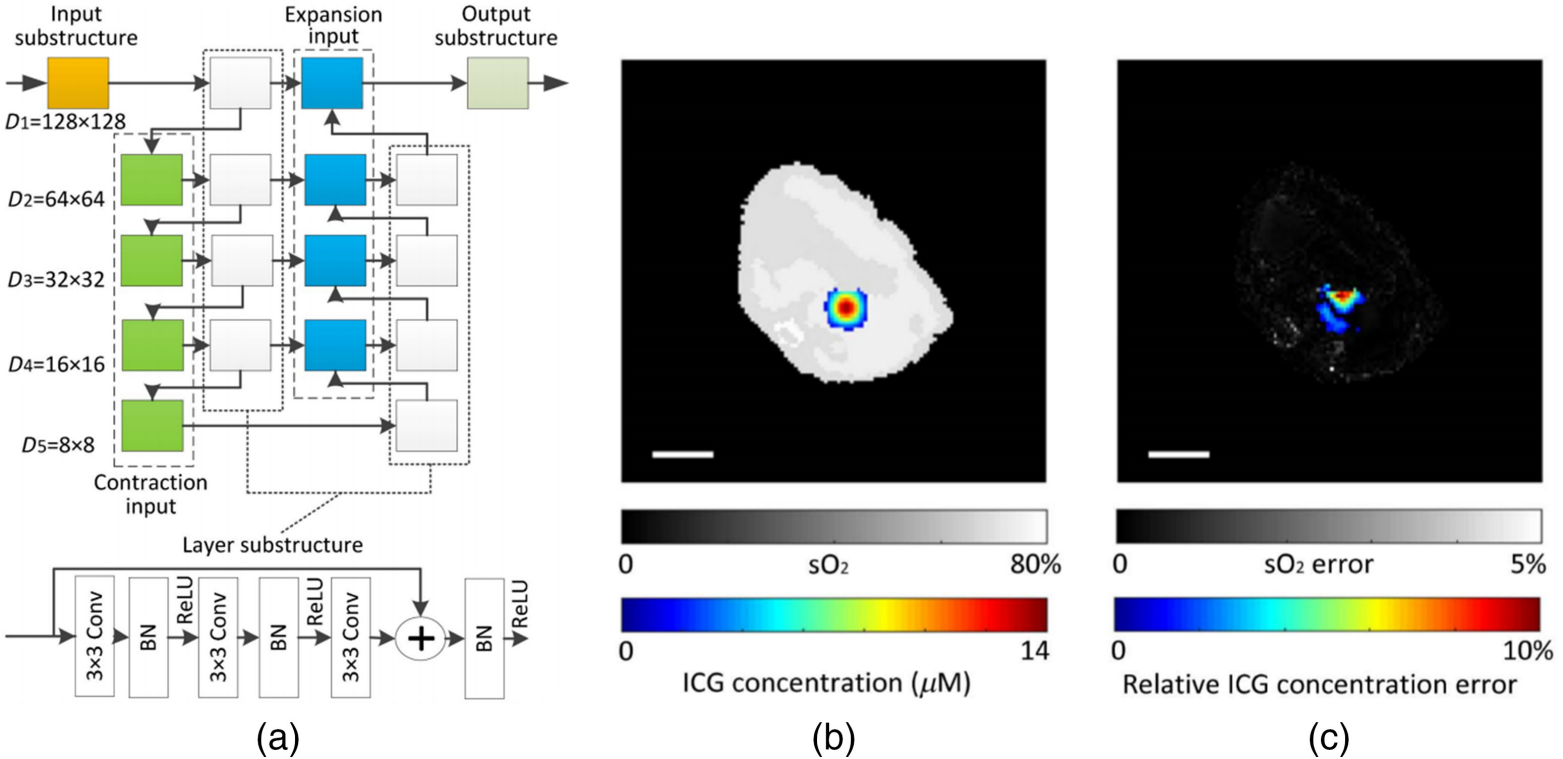
(a) The ResU-Net architecture implemented by Cai et al. (b) Simultaneously reconstructed ${\mathrm{sO}}_{2}$ map (gray) and ICG concentration (color). (c) Absolute ${\mathrm{sO}}_{2}$ error (gray) and relative ICG concentration error (color). Scale bars: 5 mm.

In 2017, Kirchner et al.^173^ used an ML method based on random forest regression to perform fluence estimation and tested its performance using simulated data. The method relied only on the local PA signal near the target and did not make use of either the global information or any feature extraction schemes such as deep NNs. In 2018, Cai et al.^35^ applied an end-to-end NN for the simultaneous imaging of oxygen saturation (${\mathrm{sO}}_{2}$) and concentration of ICG using multi-wavelength PA images as input, taking advantage of DL’s strong ability to represent complex mapping. Their network, shown in Fig. 7(a), was a modified U-Net with embedded residual structures. According to their simulation, the mean estimation errors of ${\mathrm{sO}}_{2}$ and ICG concentration were 0.76% and 3.26% for the circular phantom, and 0.51% and 7.51% for the digital mouse. The imaging result of the digital mouse is shown in Figs. 7(b) and 7(c). This model exhibited certain immunity to noise with relative error as low as 1.43% (20 dB SNR) and 16.8% (10 dB SNR). Hoffer-Hawlik et al.^123^ developed absO2luteU-Net, which used a new activation function with ELU instead of the ReLU in U-Net to calculate ${\mathrm{sO}}_{2}$ based on dual-color PA image (700 and 900 nm). The model produced an RMSE and SO-RMSE of 4.49% and 18.4%, respectively, compared to 75.5% and 64.8% by linear unmixing. AbsO2luteU-Net exhibited surprisingly low-noise sensitivity, the pixel-wise RMSE remained <6% when the SNR varied from 0 to 20 dB. Chen et al.^124^ used a U-Net with the leaky ReLU activation function to calculate optical absorption. The input of network is the reconstructed images obtained at a single wavelength, and the output is the optical absorption map. The LF used was the summation of MSE and the TV regularization term. The relative error of the outputs in different absorption backgrounds was <10%.

In addition to the simple U-Net, Yang et al. presented a combination model, called deep residual and recurrent NN, to estimate ${\mathrm{sO}}_{2}$ from the initial pressure images taken at two wavelengths.^125^ The networks used complex layers including convolution branch and residual branch in U-Net. According to simulation, the error generated by the network was as low as 1.43% compared with 62.39% by linear unmixing. Because only two wavelengths were used, the estimation process took only 18.4 ms. In 2019, Yang et al.^126^ proposed a complex autoencoder called EDA-net, which was intended specifically for QPAI to achieve accurate quantification of ${\mathrm{sO}}_{2}$ from PA images acquired at 21 wavelengths (700 to 800 nm in 5-nm steps). EDA-net had an encoder path to extract information, a decoder path to accept feature images and calculate ${\mathrm{sO}}_{2}$, and several aggregation nodes to mix information. They were built by convolutional layers. Each layer of the encoder path was connected to each layer of the decoder path by aggregation nodes. Compared with linear unmixing, ResU-Net, U-Net++,^174^ and EDA-net were superior. The authors also found that when the number of wavelengths was increased to more than nine, the mean error did not decrease significantly. In addition to this automatic encoder structure (including U-Net and its variants), Gröhl et al.^131^ used a nine-layer fully connected NN to reconstruct ${\mathrm{sO}}_{2}$ from initial pressure images with 26 wavelengths (700 to 950 nm, step size 10 nm) in 2019. The model operated in a pixel-wise manner, meaning that local information, rather than global information, was employed. For *in vivo* data of porcine brain and human forearm, the network’s output of 90% and 98% was close to the expected arterial blood oxygenation values of healthy subjects compared with linear unmixing (68% and 80%, respectively). This was the first attempt to apply DL on *in vivo* QPAI data, and the results were superior to those obtained using linear spectral unmixing.

Durairaj et al.^130^ proposed an unsupervised learning approach for PA spectral unmixing. Their model included two networks: an initialization network and an unmixing network. The weights of the initialization network were used as the initial weights for the unmixing network. The initialization network was a pixel-wise model. The number of input and output nodes was equal to the number of wavelengths (six in their case), and the number of hidden nodes was equal to the number of targets (three in their case, which were ${\mathrm{HbO}}_{2}$, ICG, and Hb). The LF was the L1 norm of the difference between the input and the output. The unmixing network had more channels and used the whole set of multi-spectral images as input. The numerical phantom had three molecular targets (${\mathrm{HbO}}_{2}$, ICG, and Hb). In terms of estimation accuracy, the model was similar to linear unmixing, but it did not need prior knowledge about the absorption spectra.

Eigenspectra multi-spectral optoacoustic tomography is a model to calculate light fluence in deep tissue.^175^ Olefir et al.^132^ combined the eigenspectra concept and DL and named the new method as DL-eMSOT. They used LSTM and CNN to calculate the weights of four spectral bases for predicting the eigenfluence. The eigenfluence of every pixel was generated by interpolation and ${\mathrm{sO}}_{2}$ was calculated by linear unmixing. In the *in vivo* experiment, they inserted a tube filled with porcine blood of known oxygenation (0% or 100%) into the animal. For most cases, DL-eMSOT outperformed eMSOT. Furthermore, the calculation speed of DL-eMSOT was 60 times faster.

For generating mask of trusted regions, Gröhl et al.^127^ presented a framework in which estimation confidences were used to increase the quantification accuracy. They used a CNR map corresponding to the initial pressure image and a U-Net network to create a joint mask. The input of the network was multi-spectral PA images and the output was a relative error map of optical absorption. It only selected the overlap regions between the two maps for evaluation to increase the quantification accuracy. To prove that their method was effective, the authors applied it to three different QPAI methods: naïve fluence correction (using a simple MC to calculate the light fluence and simulated the initial pressure), fluence correction (using U-Net to calculate the light fluence and the initial pressure), and direct absorption estimation (using U-Net to calculate the absorption coefficient directly). The results showed an accuracy increase of nearly 80% when applying a 50% confidence threshold in the direct absorption estimation model, which also produced the best results among the three models. Luke et al. presented O-net, in which two U-Nets shared the same input and were combined to estimate the vascular ${\mathrm{sO}}_{2}$ and segment the blood vessels. The input of the network was dual-wavelengths PA data.^128^ In their results, ${\mathrm{sO}}_{2}$ was estimated only in the blood vessels (as segmented by the ground-truth oxygen maps) when the network was trained. The model exhibited certain immunity to noise and produced absolute errors of 5.1% and 13.0% when the SNR was 25 and 5 dB, respectively. Bench et al. used two simple U-Nets for the segmentation and ${\mathrm{sO}}_{2}$ estimation of 3D PA images.^176^ The network was trained on a data set generated numerically using 3D vessel models acquired from CT scans of human lung vessels. In the simulation, a skin model consisting of the epidermis, dermis, and hypodermis layers with different optical properties was applied. They used the segmentation results to generate a mask to calculate the mean ${\mathrm{sO}}_{2}$ and found the mean difference between the ground truth and the output to be 0.3%, with a standard deviation of 6.3%.

## 4 Discussion

### 4.1 Network Architecture and LF

As discussed earlier, the information loss due to deficient signal detection has been a major roadblock for the development of PAI.

According to the published results, we find that: (1) U-Net is almost a panacea that has been applied in most of the tasks; (2) the application of DL in the traditional reconstruction frameworks usually generates better results than other approaches; and (3) the better results are often accompanied by increased network complexity, such as more branches,^36^^,^^126^ more connections,^86^^,^^87^ and more complex layers or blocks.^107^^,^^149^

Is there a more fundamental design principle? Can we design networks based on physics and generate interpretable structures that correspond to the first-principal process? Since interpretability is associated with the trustworthiness, expandability, and sustainable development of the DL approaches, complementing the data-driven approaches with explainable ingredients or top-down designs has been a major endeavor for current and future research.^177^

Supplementing the missing information is also one of the major aims of network design. Combining DL with traditional reconstruction methods is a good choice, which has yielded good results in all types of task.^68^^,^^109^^,^^110^^,^^132^ Among them, applying DL in iterative model-based methods to calculate the update or the regularization term has been the most successful, probably because it can best leverage the prior information about the PA physics and the imaged object. In addition, the iterative method is more robust than other models (including preprocessing model, postprocessing model, and direct reconstruction model).^112^

People often use MSE as a common LF, but more items (such as SSIM, PSNR, and PC^99^^,^^103^) can be added for various purposes such as better information extraction and faster convergence. Notably, GAN is an emerging network^103^^,^^107^ whose LF contains a network that is being simultaneously trained. The GAN network has been shown to recover fine details better.

Real-time data processing and display are important for clinical applications, and currently most PAI systems are based on personal computers or FPGAs. Limited by the available computing resources, people have to pay close attention to the computational complexity and the overall size when designing the network. The exploratory works in the CV field, such as SqueezeNet,^178^ MobileNet,^179^ and ShuffleNet,^180^ may be inspiring for the development of future DL-assisted PAI systems with small size and high speed.

### 4.2 Data Set

The availability of high-quality data set is of paramount importance to the success of the DL methods. A prominent example is the development of CNNs, which were made possible by ImageNet.^181^ However, PAI is an emerging technology lacking high-quality data sets. The following remedies were used in the PAI community to alleviate the problem.

• *Using simulated data.* It is a common method to build data sets, which is covered in Sec. 2.2.2.
• *Data augmentation.* It can be used to generate more data from the existing data. Common methods include random pixel shifting, rotation, cropping, warping, vertical and horizontal flipping, and adding noise.^70^^,^^72^
• *Transfer learning*. It is a commonly used technique, in which the network is pretrained on public/simulated data before being retrained on a more relevant, high-quality data set with limited size.^70^^,^^112^
• *Network downsizing*. It can be used to reduce the input size. For example, when the input is cropped from 256×256 to 64×64, the size of the data set is equivalently increased.^100^^,^^101^ In addition, it can reduce the number of network parameters so that the network can be trained with fewer data.
• *Unsupervised model*. It is an alternative option where no ground truth is needed.^130^

Despite the above-mentioned solutions, large, high-quality PA data sets are bound to facilitate the development of DL-PAI. On the one hand, a high-quality data set makes network development more efficient, reliable, and flexible. On the other hand, there is no effective way to compare the performance of different models. This is to a large extent because the systems and methods used by different research groups are vastly diverse, and intermural standards for the imaging data and evaluation metrics do not exist. Most importantly, building a high-quality and sizable database tailored for PAI is necessary. Fortunately, some organizations have begun building such data sets.^63^

Currently, building data sets and applying them in real-world applications remain challenging. Since DL models are unexplainable, one is unsure whether a model is solely dependent on the physics of image formation, or it is also affected by specific image features. One must be aware that once the network parameters are coupled with image features, it can hallucinate based on what it has learnt, so one must confirm the result by comparing it with the ground truth. Because the image features of PA are unique, it is difficult to obtain a gold standard image using other modalities such as MRI, and without such ground-truth knowledge, how can we know that the network is working properly? This is especially true for QPAI, since PAI is currently the only imaging modality for ${\mathrm{sO}}_{2}$ and chromophore concentration measurement (with high spatial resolution in great depth). Thus due to the lack of a reliable QPAI computation method, it is currently impossible to obtain the ground truth in live animals or humans. Developing realistic phantoms for PAI and QPAI is thus an important future research direction.

In real applications, data balancing is also noteworthy. This means that the training set distributions will affect the predicted results.^182^^,^^183^ It is a general rule of thumb that the proportion of each data type is consistent with that in the actual application. If the condition is unsatisfied, applying weight parameters is a useful method to balance the data.^69^

### 4.3 Reliability of Results

As we have briefly discussed in Sec. 4.2, in what condition, and to what extent can we trust an image reconstructed by DL methods? For example, we have shown that DL can be used to improve the image quality under limited-view detection. Since in the missing cone all information is completely lost, what the NN does is to fill in the lost information as in super-resolution, rather than to amplify weak frequency components as in deconvolution. Since a physical mechanism for super-resolution is lacking (such as introducing non-linearity into the image formation process), the true reason for the recovery of the missing frequency components may be that the network recognizes the image features during training. This means that a network trained with images acquired at the liver region would produce unrealistic features in the kidney by hallucinating. We expect that even for the imaging of the same organ (e.g., the breast), the network may produce fake results if the target being imaged has features that are new to the network. The situation will become worse when a network trained on one system is translated to another, especially when the two systems have different types of probes and/or imaging targets. Thus the study and verification of the generalizability of the DL networks are important.

Tradition methods also perform well on promoting the PA image quality under non-ideal detection.^184^^,^^185^ Generally speaking, they are currently more reliable than the DL methods. Comparison between and integration of the traditional methods and the DL methods are important future research directions.

### 4.4 Applications

Compared with the traditional modalities, such as CT, US, and MRI, PAI is an emerging imaging modality, in which most clinicians do not have much experience. It is easier for doctors to accept PAI if DL can be used to extract key image features or relevant physiological parameters for auxiliary diagnosis.

For some clinical applications, information loss is inevitable. For hand-held probes, it is difficult for common ultrasonic medical probes to receive wide-band PA signals (e.g., 0.1 to 10 MHz). Shallow features are prone to be affected by the sparse sampling, whereas the deep features are more subject to limited view. In these situations, DL is an effective means to compensate for the lost information. DL can also facilitate the development of low-cost and portable equipment, which usually have reduced image quality. In such applications, building the data set is comparatively easier and DL will play an important role. For example, one can get high-quality images using powerful lasers as the ground truth.

In addition to providing information about vascular morphology,^186^ a unique capability provided by PA is the imaging of chromophore concentrations and ${\mathrm{sO}}_{2}$ in tissue. Due to the lack of a closed-form solution in these problems, data-based approaches (not limited to DL) have achieved promising results.^175^ We expect DL to play an increasingly important role in QPAI.

Specific devices incorporating DL can achieve good imaging results unachievable otherwise.^115^ It is believed that, combined with DL, novel PAI devices can be designed to meet the needs of various applications. In particular, as 3D PAI is becoming increasingly popular,^65^^,^^72^^,^^104^^,^^129^ using DL for 3D reconstruction, generating 3D images from 2D data, or processing 3D images are important future directions.

### 4.5 Conclusion

Despite the above challenges and difficulties, we envision that DL will continue to make great impact for PA imaging. The unparalleled capability of DL in information extraction, fusion, and high-speed processing is bound to bring PAI new vigor and opportunities. This review is thus not a conclusion, but rather the herald of the exciting era of DL-based PA imaging.
